# Preliminary evidence of localizing CD8+ T-cell responses in COVID-19 patients with PET imaging

**DOI:** 10.3389/fmed.2024.1414415

**Published:** 2024-05-15

**Authors:** Hans J. P. M. Koenen, Ilse J. E. Kouijzer, Michel de Groot, Steffie Peters, Daphne Lobeek, Evelien A. J. van Genugten, Dimitri A. Diavatopoulos, Nienke van Oosten, Sanne Gianotten, Mathias M. Prokop, Mihai G. Netea, Frank L. van de Veerdonk, Erik H. J. G. Aarntzen

**Affiliations:** ^1^Department of Laboratory Medicine, Radboud University Medical Center, Nijmegen, Netherlands; ^2^Department of Internal Medicine and Radboud Center for Infectious Diseases, Radboud University Medical Center, Nijmegen, Netherlands; ^3^Department of Medical Imaging, Radboud University Medical Center, Nijmegen, Netherlands

**Keywords:** SARS-CoV2, PET imaging, mucosal immunity, CD8+ T-cells, trafficking

## Abstract

The upper respiratory tract (URT) is the entry site for severe acute respiratory syndrome-coronavirus-2 (SARS-CoV-2), from where it further disseminates. Early and effective adaptive immune responses are crucial to restrict viral replication and limit symptom development and transmission. Current vaccines increasingly incorporate strategies to boost mucosal immunity in the respiratory tract. Positron emission tomography (PET) is a non-invasive technology that measures cellular responses at a whole-body level. In this case series, we explored the feasibility of [^89^Zr]Zr-crefmirlimab berdoxam PET to assess CD8+ T-cell localization during active COVID-19. Our results suggest that CD8+ T-cell distributions assessed by PET imaging reflect their differentiation and functional state in blood. Therefore, PET imaging may represent a novel tool to visualize and quantify cellular immune responses during infections at a whole-body level.

## Introduction

The upper respiratory tract (URT) represents the site of entry for severe acute respiratory syndrome coronavirus 2 (SARS-CoV-2) (1). In the absence of an adequate mucosal immune response, including T-cells (2), it may replicate and spread to the lower respiratory tract (LRT) and eventually distant organ sites (3). Mucosal T-cells help control the viral load (4) and limit the (progression of) disease, thus reducing viral spread in the population (5). CD8+ T-cells are major effector cells of these local cytotoxic responses to viral infection and are rapidly recruited to the nasopharynx following a controlled challenge of volunteers with SARS-CoV-2 (6). Although CD8+ T-cells are important for the clearance of infected cells, delayed and persistent bystander activation of CD8+ T-cells in hospitalized patients suggests that they may also contribute to lung pathology (7, 8).

Little is known about the spatial distributions of CD8+ lymphocytes at a whole-body level (9) during acute coronavirus disease 2019 (COVID-19). This case series describes the *in vivo* distribution of CD8+ T-cells in hospitalized patients during active SARS-CoV-2 infection using positron emission tomography (PET) imaging and functional characterization of circulating lymphocytes using flow cytometry.

## Methods

### Study design

A prospective observational, open-label, non-randomized pilot study was performed on patients admitted to the hospital with active SARS-CoV-2 infection. Eligibility criteria included age > 50 years and PCR-confirmed SARS-CoV-2 infection. Patients underwent a [^89^Zr]Zr-crefmirlimab berdoxam PET/CT scan; 20 mL EDTA blood and 10 mL EDTA plasma were collected immediately before tracer injection. Patients were monitored, and vital signs were measured every 6 h during admission. The protocol was approved by the Institutional Review Board, and all patients provided written informed consent (ClinicalTrials.gov identifier NCT04874818). In total, 6 patients were counseled for this study, 4 were eligible, and three patients underwent scanning. One patient did provide informed consent but was not scanned as a tracer dose was not available. Two patients did not provide informed consent due to claustrophobia (*n* = 1) and radiation dose (*n* = 1). As the SARS-CoV-2 pandemic resided, further enrollment was halted, and the data were preliminarily analyzed.

### Positron emission tomography imaging

[^89^Zr]Zr-crefmirlimab berdoxam is a 79,946 Da minibody (Mb) directed to the CD8 antigen, conjugated with deferoxamine (Df) and radiolabeled with ^89^Zr for imaging CD8+ cells in humans. The total molecular weight of the [^89^Zr]Zr-crefmirlimab berdoxam imaging agent is 81,453.8 Da. It binds to both CD8αα and CD8αβ, thus binding to mature T-cells, developing thymocytes, TCRαβ-expressing gut intra-epithelial T-cells, some γδT-cells, and some natural killer and dendritic cell subsets. The lack of Fc-receptor interaction domains makes it pharmacologically inert with respect to Fcγ-receptor-mediated effector functions. [^89^Zr]Zr-crefmirlimab berdoxam was produced according to Good Manufacturing Practice as described previously (10, 11) and obtained from ImaginAb Inc. A single dose of 37 MBq ± 10% (range 37 to 39.3 MBq) of [^89^Zr]Zr-crefmirlimab berdoxam (total minibody mass of 1.5 mg) was administered as a slow bolus intravenously over 5 min. No premedication was administered. At 21–27 h post-injection, PET acquisitions were performed from the skull to the greater trochanter on a Siemens Biograph mCT (Siemens Healthineers, Knoxville, United States), using 5 min per bed position for the chest and 3 min per bed position for the remainder. Images were reconstructed into a 200 × 200 matrix TrueX+TOF (21 subsets; 3 iterations). A low-dose CT scan without iodinated contrast was used for anatomical reference and attenuation correction. Volumes of interest were drawn manually to compute maximum and mean standardized uptake values (SUV_max_ and SUV_mean_, respectively). For the individual organ sites, tracer uptake values are expressed as tissue-to-blood ratio (TBR) calculated as the SUVmean of the tissue divided by the SUVmean of the blood pool, measured in a spherical volume-of-interest of at least 10 mm diameter in the descending aorta.

### Flow cytometry

A 10-color flow cytometry of freshly drawn blood samples was performed and analyzed as described previously (12). The following monoclonal antibodies were used: CD57-FITC, CD45RA-ECD, CD8APC-AF750, CD45-KO (Beckman Coulter), CD196-PE, CD194-PC7, CD199-AF488, CD25-APC (BD Biosciences) CD183-PerCpCy5.5, CD197-BV421, KLRG1-PerCpCy5.5 (Biolegend), CD4-AF700, CD279-PC7 (eBioscience), and CD28-PE (Dako). Two 10-color panels were used; panel 1 included CD45RA, CD196, CD8, CD183, CD194, CD25, CD4, CD199, CD197, and CD45 and panel 2 included CD57, CD28, CD45RA, KLRG1, CD279, CD25, CD4, CD8, CD197, and CD45.

### Staining

All cells were surface stained in 25 μL of surface staining master mix at RT for 20 min. The cells were washed twice by adding PBS + 0.2% bovine serum albumin (BSA) and centrifuged at 250 x*g* for 2.5 min. The buffer was removed by flicking the plates. Before acquisition, whole blood-derived cells were resuspended in 100 μL PBS + 0.2% BSA.

For intracellular staining, the surface-stained peripheral blood mononuclear cells (PBMC) were fixed and permeabilized using the Fix/Perm solution (eBioscience, Vienna, Austria). After 30 min at 4°C, protected from light, the cells were washed and centrifuged at 250 xg for 2.5 min twice using a permeabilization buffer (eBioscience, Vienna, Austria). Then, 25 μL of the intracellular staining master mix was applied, and the samples were incubated for 30 min at 4°C, protected from light. After a second washing step using permeabilization buffer, the cells were resuspended in 100 μL PBS + 0.2% BSA for data acquisition.

### Gating strategy

Each sample was analyzed by two multi-color antibody panels, as described previously. For each panel, the single cells, identified by plotting the FS Time Of Flight (FS TOF) against FS, within the leukocyte (CD45+) population were first gated, and then the lymphoid cells were gated.

In both panels, the lymphocytes were discriminated by forward scatter and side scatter. Within the CD8 + CD4- cells, maturation stages were defined based on CD45RA and CCR7 expression, namely, CD45RA+/CD197+ naïve cells, CD45RA-/CD197+ central memory (CM) cells, CD45RA-/CD197 effector memory (EM) cells, and CD45+/CD197- terminally differentiated effector memory (TEMRA) cells. Within these T-cell maturation stages, the percentage of CD196, CD183, CD194, and CD199 (panel1) and CD57, CD28, KLRG1, CD279, and CD25 (panel 2) expressing cells was determined.

### Flow cytometry measurements and data analysis

Data were acquired using a Navios Flow Cytometer, as described above. Each sample suspended in 100 μL was measured for 60 s, representing 75% of the sample volume. This prevented the intake of air, leading to a non-specific signal at the end of the measurement. For the flow cytometry analysis, a manual gating strategy was conducted. Each analysis was verified by two independent specialists to prevent gating errors. Analyzed data were stored batch-wise per 20 samples each. The statistics were exported batch-wise for further analysis.

## Results

### Patient characteristics

The study was performed in an acute clinical setting where patients were admitted with respiratory symptoms and PCR-test positive for SARS-CoV-2. During the period of subject enrollment in February and March 2022, the SARS-CoV-2 delta-variant BA.1 and BA.2 were dominant in The Netherlands.^1^ However, the determination of virus variants and exact viral load was not routinely performed and is not available for this cohort.

Case 1 was a 79-year-old man with chronic obstructive pulmonary disease (COPD) who was admitted to the hospital with respiratory stress 4 days after the onset of COVID-19 symptoms. He had not been previously vaccinated. PCR testing of the nasopharyngeal swab for SARS-CoV-2 on admission was positive, with a cycle threshold of 16. The oxygen saturation level was 94% without additional oxygen needed. Laboratory evaluation showed lymphopenia (0.44 × 10^9^/L, normal values 1.00–3.50 × 10^9^/L) and a C-reactive protein level of 17 mg/L (normal values <5 mg/L). Treatment with prednisone 30 mg once daily was initiated under suspicion of exacerbation of COPD triggered by SARS-CoV-2 infection. PET/CT imaging was performed on day 7 after the onset of symptoms, and the patient was discharged from the hospital 6 days after admission.

Case 2 was an 83-year-old man with type 2 diabetes, hypertension, and chronic idiopathic thrombocytopenic purpura admitted to the hospital with respiratory stress and worsening of known late-onset cerebellar ataxia 2 days after the onset of symptoms. He was vaccinated twice against SARS-CoV-2 with BNT162b2 (Tozinameran) and received a booster vaccination with the same vaccine before admission. On admission, PCR testing of the nasopharyngeal swab for SARS-CoV-2 was positive, with a cycle threshold of 20. The oxygen saturation level was 96% without additional oxygen needed. Laboratory evaluation showed normal lymphocyte counts (1.34 × 10^9^/L, normal values 1.00–3.50 × 10^9^/L) and a C-reactive protein level of 70 mg/L. Treatment with intravenous immunoglobulin and donor-platelet infusion was initiated because of thrombocytopenia. PET/CT imaging was performed on day 5 after the onset of symptoms, and the patient was discharged from the hospital 8 days after admission.

Case 3 was an 89-year-old man with COPD and cardiovascular disease admitted to the hospital with respiratory stress 10 days after the onset of symptoms. He was vaccinated with BNT162b2 (Tozinameran) and had received a booster vaccination with the same vaccine prior to admission. PCR testing of the nasal swab for SARS-CoV-2 was positive, with a cycle threshold of 23. Oxygen saturation was 90% with 4 L/min oxygen. Laboratory evaluation showed lymphopenia (0.22 × 10^9^/L, normal values 1.00–3.50 × 10^9^/L) and a C-reactive protein level of 81 mg/L. Treatment with dexamethasone 6 mg once daily was initiated. PET/CT imaging was performed on day 13 after the onset of symptoms, and the patient was discharged 22 days after admission.

### Visualizing CD8+ T-cells during early and late stages of COVID-19

**Figure 1:**
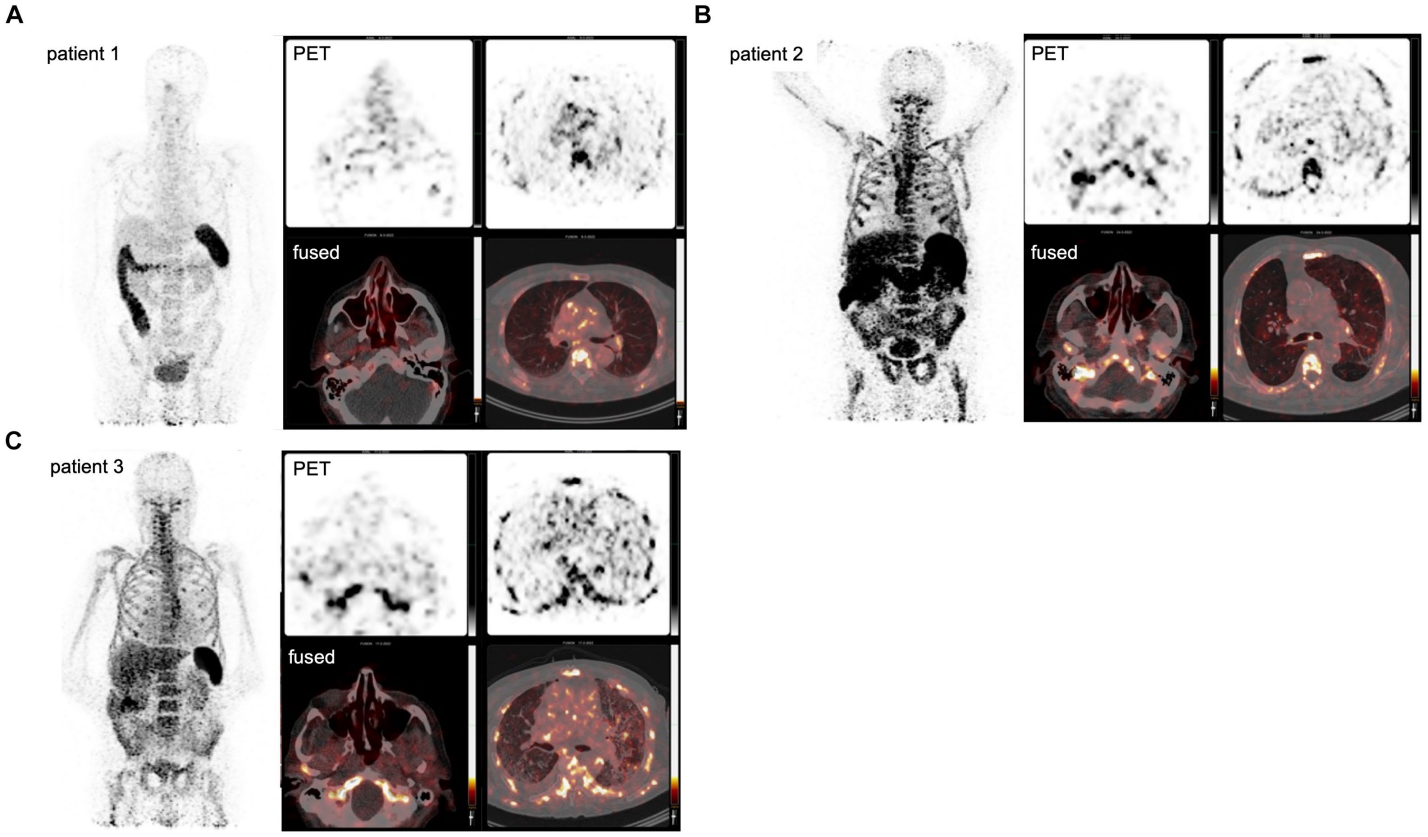
PET imaging visualizes the *in vivo* distribution of CD8+ T-cells in acute COVID-19. Using a Zirconium-89-labeled CD8α chain targeting minibody for PET/CT images, the *in vivo* distribution of CD8+ T-cell was visualized at a whole-body level in three subjects with acute COVID-19 **(A–C)**. The left-upper panel displays the maximum intensity projections (MIP), the right panels display the PET-only transversal view of the upper respiratory tract (upper-middle panel) and lower respiratory tract (upper-right panel), and PET/CT fused transversal view (lower panels). CD8+ T-cell-rich organs, e.g., spleen and bone marrow, show the highest uptake and activity in the excretory organs, such as the hepatobiliary tract, resulting in bowel activity. In patients 1 and 2, the uptake in the nasal mucosa is markedly increased (arrows).

**Figure 2:**
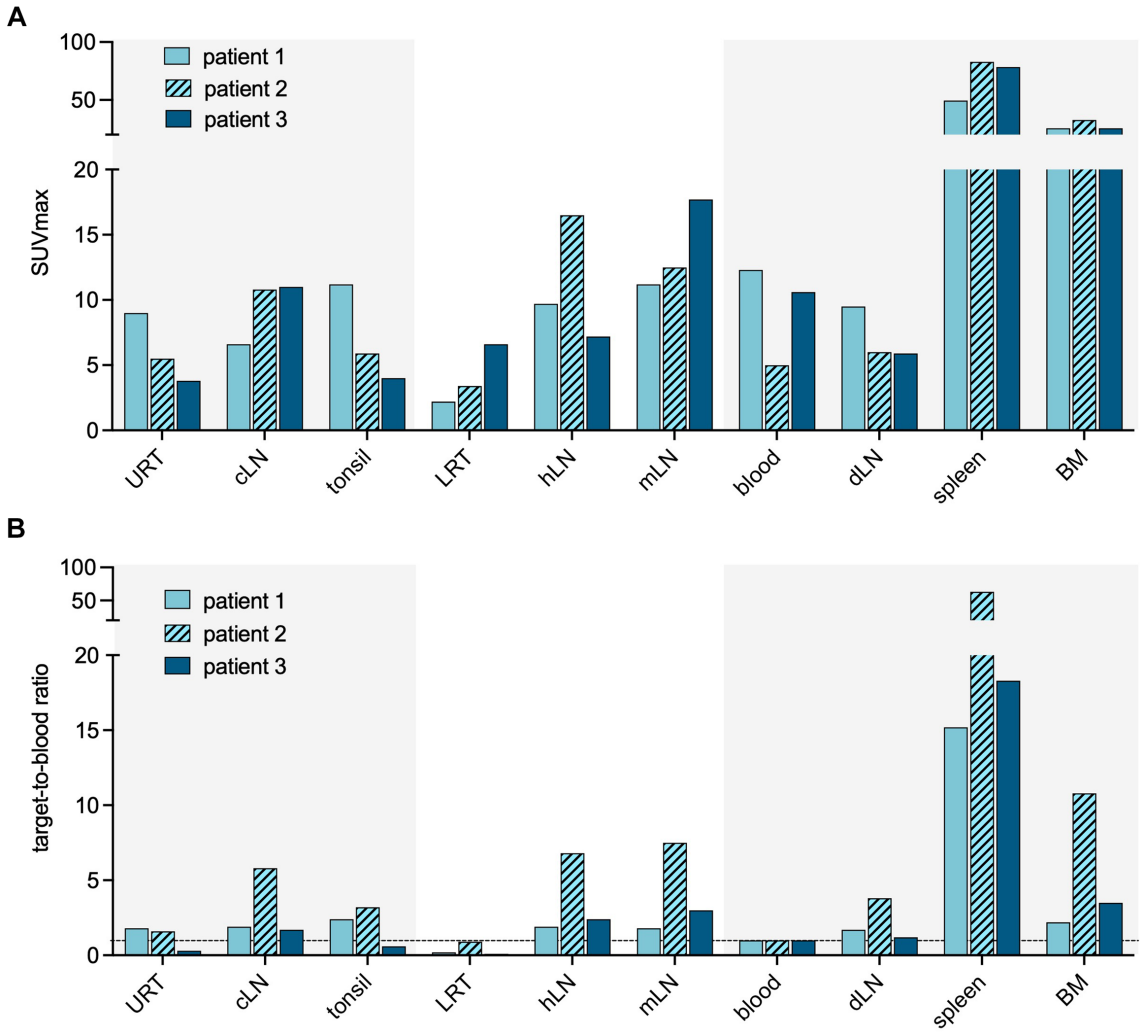
Quantification of CD8 targeting PET signal in acute COVID-19. Quantification of the PET signal in either maximum standardized uptake value (SUV_max_) **(A)** or target-to-blood ratios **(B)** for the upper respiratory tract (URT), cervical lymph nodes (cLN), tonsils, lower respiratory tract (LRT), hilar lymph nodes (hLN), mediastinal lymph nodes (mLN), spleen, and bone marrow (BM).

High tracer accumulation is commonly observed in CD8+ T-cell-rich organs, such as the spleen and bone marrow, as well as activity in the excretory organs, such as the hepatobiliary tract, which subsequently results in bowel activity (10, 11). Patients 1 and 2 were imaged at early time points after the onset of symptoms, on days 7 and 5, respectively. Both patients presented with mild symptoms and were discharged soon after imaging. PET/CT imaging showed an increased presence of CD8+ T-cells in the nasal mucosa in these patients, compared to patient 3 (TBR 6.8 and 4.3 vs. TBR 1.4, respectively) (Figures 1, 2). A similar pattern was observed in the URT-associated lymphoid tissue, e.g., tonsils (TBR 7.4 and 5.3 vs. TBR 1.4) and cervical lymph nodes (TBR 13.4 and 3.1 vs. TBR 3.9). No tracer uptake was observed in the lower respiratory tract in patients 1 and 2 (Figures 1, 2).

Patient 3 presented with dyspnea and increased oxygen demand, suggesting involvement of the lower respiratory tract. This patient was imaged on day 10, and no tracer uptake in the URT was observed (Figure 1). Although absolute tracer uptake in the affected lung parenchyma was increased (SUV_max_ 6.6) as compared to patients 1 and 2 (SUV_max_ 3.4 and 2.2), TBR was in the same range (TBR 2.4 vs. 4.2 and 1.1) (Figure 2), indicating the mere presence of tracer in the increased blood volume in the affected parenchyma, rather than trans-endothelial migration of CD8+ T-cells into the interstitial space.

In addition to the distribution patterns in the respiratory tract, patient 1 also showed markedly increased presence of CD8+ T-cells across primary (bone marrow and spleen) and distant secondary (inguinal and mediastinal lymph nodes) lymphoid organs, as well as other organ sites, including the liver, kidney, and gluteal muscle.

### PET-based distribution patterns correspond to CD8+ T-cell phenotype

**Figure 3:**
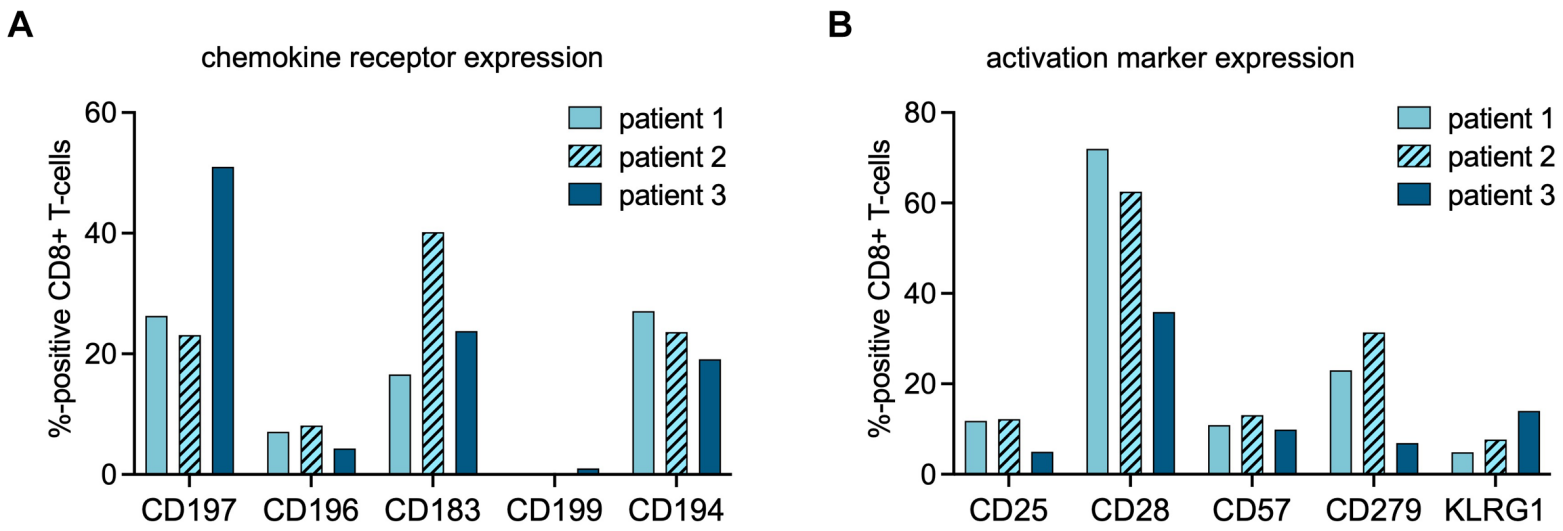
Flow cytometric analysis of CD8+ cells at time point of PET imaging. The percentages of CD8+ cells expressing chemokine receptors **(A)** or activation markers **(B)** at the time point of PET imaging were assessed using multipanel flow cytometry (chemokine receptor profiles: CD197 = CCR7, homing to secondary lymphoid organs; CD196 = CCR6, homing to mucosal tissues; CD183 = CXCR3, a general leukocyte trafficking receptor; CD199 = CCR9, homing to gastrointestinal organs, CD194 = CCR4, Th2 T-cell trafficking). (activation marker profiles: CD25 = interleukin (IL)-2 receptor; CD57 = human natural killer-1 (HNK1), marker of immune aging; CD279 = programmed death receptor (PD)-1; CD28 = co-stimulatory receptor, KLRG-1 = Killer cell lectin-like receptor subfamily G member 1, a co-inhibitory receptor on late-differentiated T-cells).

The increased TBR in the URT in patients 1 and 2 coincided with higher expression of C-C chemokine receptor type 6 (CCR6/CD196) on the peripheral blood total CD8+ cells, in comparison to patient 3 (8.1 and 7.1% vs. 4.3%) (Figure 3).

Patient 2 showed increased TBR in primary, distant secondary lymphoid organs, and non-lymphoid organs, suggestive of rapid in- and efflux of CD8+ T-cells from the circulation. In this patient, the fraction of C-X-C motif chemokine receptor 3 (CXCR3/CD183) positive lymphocytes among the total CD8+ population was higher than in patients 1 and 3 (Figure 3).

**Figure 4:**
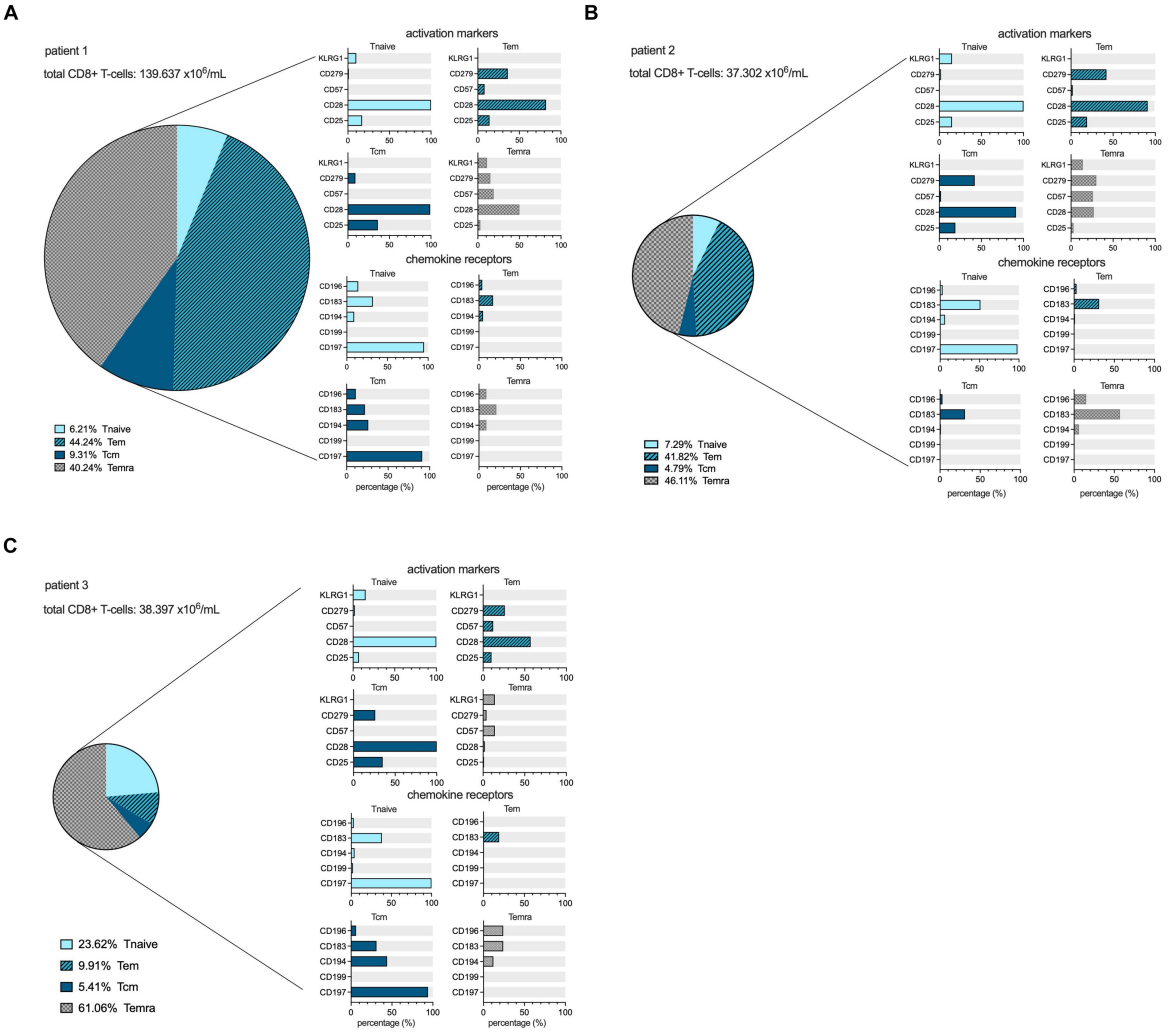
CD8+ cell maturation stages and flowcytometric subtyping. Distribution of different maturation stages, based on differential expression of CD45RA and CD197, within the CD8+ lymphocyte compartment in the peripheral blood at the time point of scanning for each patient in percentages, as well as the expression profiles of activation markers and chemokine receptors among the T_naive_ (light blue), T_effector memory_ (dark blue, hatched), T_central memory_ (dark blue filled), and T_emra_ (grey hatched) populations within CD8+ T-cells. The size of the pie chart corresponds to the absolute number of CD8+ T-cells (in ×10^6^/mL) for each subject **(A–C)**.

In both patients 1 and 2 imaged earlier after the onset of symptoms, the CD8+ T-cell population was dominated by an abundance of CD45RA-/CD197+ effector memory (Tem) and CD45RA+/CD197- terminally differentiated effector memory (Temra) phenotypes, indicative of recent priming (Figure 4). At a later stage of infection in patient 3, the CD8+ T-cell population was characterized by an increase in Temra phenotypes, as well as CD45RA+/CD197+ naïve T-cells (Figure 4), suggestive of prolonged antigen stimulation and a replenished CD8+ T-cell reservoir. In addition to the evidence of evolving CD8+ T-cell differentiation, patient 3 also had higher frequencies of senescent/exhausted CD8+ T-cells as indicated by the loss of expression of CD28 (35.9% vs. 62.5 and 72.0%) and reduced programmed death receptor-1 (CD297) expression (6.9% vs. 31.4 and 23.0%) among the total CD8+ population (Figure 3).

## Discussion

Cellular immune responses are important for viral clearance and limitation of disease severity of SARS-CoV-2 infection. CD8+ T cells can help restrict viral replication, disease, and transmission (2, 13) but may also contribute to pathology (7, 8). An early and balanced recruitment and activation of CD8+ T cells in the mucosa and their expansion in secondary lymph nodes is a complex process that involves a concerted response of multiple immune cell populations and inflammatory mediators (13, 14). Previous studies highlighted that T-cell migration in response to inflammatory stimuli is governed by cell-intrinsic and cell-extrinsic factors, which vary throughout stages of differentiation and organ sites (15). This results in a spatial and temporal compartmentalization of T-cell subsets (16), which is difficult to infer from peripheral blood samples, which contain only a fraction of the total T-cell population (17). This study responds to the need for technologies that allow evaluation of the presence and dynamics of T-cells on a whole-body scale. PET imaging meets these prerequisites as it provides quantitative data in a non-invasive fashion and is feasible in clinical studies. We employed a radiolabeled minibody targeting the human CD8α subunit (10, 11) to interrogate the *in vivo* distribution of CD8 T-cells in patients admitted to the hospital with COVID-19.

Albeit in a small series of patients, PET imaging demonstrated the differential distribution of CD8+ T-cells in the mucosa and associated lymphoid organs of the URT during early and later stages of SARS-CoV-2 infection, underscoring the concept of spatial and temporal compartmentalization of CD8+ T-cell responses to respiratory viral infection. Furthermore, the observed differences in patterns of CD8+ T-cell distribution across mucosa, primary and secondary lymphoid organs, blood pool, and peripheral tissues can be correlated with changes in CD8+ T-cell functional phenotypes. The chemokine receptor CXCR3 generally regulates leukocyte trafficking, promoting T-helper 1 recruitment and maturation (18). CXCR3 expression was the highest in patient 3, coinciding with the highest tracer uptake values across both lymphoid and non-lymphoid organs, suggesting increased trafficking of CD8+ T-cells from the circulation into peripheral tissues. Furthermore, the expression levels of the chemokine receptor CCR6, which directs T-cells to mucosal tissues in response to its ligand macrophage inflammatory protein 3 alpha (MIP-3α/CCL20) (18), were the highest in the two patients scanned earlier during their course of the disease and were associated with the highest tracer uptake values in the nasal mucosa. Finally, in the patient with a prolonged course of disease and features of exhaustion and senescence, predominantly in the circulating terminally differentiated CD8+ lymphocyte compartment, the lowest tissue-to-blood ratios were observed. This finding is consistent with the limited capacity of exhausted CD8+ T-cells to infiltrate into tissues (19). These observations hint at the potential of PET/CT imaging to develop quantitative parameters inferred from the spatial and dynamic substrates of CD8+ T-cell behavior.

It is important to note that these cases provide anecdotal observations and warrant further studies that include translational data linking functional or phenotypic characterization of CD8+ T-cells to tracer distribution patterns observed on PET imaging. In general terms, CD8+ T-cell responses in elderly patients, as in this case series, may be impaired by reduced clonal diversity (20) and proliferative capacity (21) and increased exhaustion (22), also shown to be relevant in SARS-CoV2 infection. It is yet unknown how these aspects of CD8+ T-cell behavior *in vivo* affect the distribution and quantification of [^89^Zr]Zr-crefmirlimab berdoxam. The limited number of subjects in this study, sharing general characteristics such as age, gender, and SARS-CoV2 variant, preclude assumptions of a direct correlation with PET imaging findings. In this respect, studies using this tracer in healthy volunteers under steady-state conditions are not available as references. Such studies are currently only feasible on ultra-sensitive PET systems (23) that allow for reducing the administered dose of Zirconium-89 to match the ICRP62 risk category IIIa, balancing the acquisition of knowledge regarding serious disease with additional effective doses by study-related radiological procedures. Furthermore, a robust analytical framework for interpretation that allows for quantitative correlation between the high-dimensional data from flow cytometry on a selected fraction of CD8+ T-cells from the blood compartment and the spatial information described by PET parameters is to be developed. The simple linear associations presented in this article should be interpreted as illustrative, aiming to stimulate researchers in immunological domains to embrace imaging technology in addition to established assays.

However, acknowledging the importance of early and local control of respiratory viruses by the adaptive immune system, a key objective of new vaccines is to develop strategies that induce robust mucosal cellular responses (24, 25). Biomarker technologies that allow early and quantitative assessment of changes in CD8+ T-cell distributions, e.g., from lymphoid compartments to mucosal linings, may provide alternative endpoints that can accelerate the development of effective vaccination approaches. Similarly, novel treatment strategies in immune-mediated inflammatory conditions that are dominated by CD8+ T-cells (26) or in which CD8+ T-cells are therapeutically targeted may benefit from non-invasive imaging approaches. The observations made in this case series highlight PET imaging with immune cell-specific tracers as an imaging biomarker that may complement current immunological assays with information on the spatiotemporal distributions of CD8+ T-cells on a whole-body scale, assessing yet another aspect of CD8+ T-cell biology (9).

In conclusion, PET/CT imaging with a radiolabeled minibody targeting CD8α on T-cells allows the localization of CD8+ T-cell responses *in vivo* in COVID-19 patients.

## Data availability statement

The original contributions presented in the study are included in the article/supplementary material, further inquiries can be directed to the corresponding author.

## Ethics statement

The studies involving humans were approved by Medisch Ethische Commissie Arnhem en Nijmegen. The studies were conducted in accordance with the local legislation and institutional requirements. The participants provided their written informed consent to participate in this study.

## Author contributions

HK: Conceptualization, Formal analysis, Writing – review & editing, Validation. IK: Writing – review & editing, Investigation. MG: Writing – review & editing, Project administration, Resources. SP: Project administration, Writing – review & editing, Methodology. DL: Methodology, Project administration, Writing – review & editing. EG: Methodology, Writing – review & editing, Formal analysis. DD: Formal analysis, Writing – review & editing, Conceptualization. NO: Writing – review & editing, Project administration, Resources. SG: Writing – review & editing, Investigation. MP: Writing – review & editing, Methodology. MN: Writing – review & editing, Formal analysis. FV: Formal analysis, Writing – review & editing. EA: Formal analysis, Writing – review & editing, Conceptualization, Writing – original draft.

## Footnotes

^1^ https://www.rivm.nl/en/coronavirus-covid-19/current/variants
